# Optimal intrathecal morphine dosing for revision hip arthroplasty: an observational study

**DOI:** 10.1111/anae.70305

**Published:** 2026-07-21

**Authors:** Shalini Patel, Vassia Paschou, Jane Quinlan, Ben Kendrick, Nicolas Suarez

**Affiliations:** ^1^ Oxford University Hospitals NHS Foundation Trust Oxford UK

Around 2–10% of primary total hip arthroplasties fail and require revision due to: infection; component wear; loosening; dislocation; persistent pain; or periprosthetic fracture [1]. The rise in life expectancy has contributed to an increasing demand for revision procedures. Revision surgery is more complex than primary surgery, involving greater surgical trauma; increased blood loss; and less predictable improvements in pain and function. However, there is little information on optimal anaesthetic and analgesic strategies.

The PROSPECT guidelines for primary total hip arthroplasty recommend intrathecal morphine to improve pain control and reduce postoperative opioid requirements [2]. However, this may increase adverse effects and risk. Whilst 100–200 μg intrathecal morphine appears effective and safe in primary total hip arthroplasty, evidence in revision surgery is limited [3]. Our study evaluated analgesic efficacy and adverse effects of intrathecal morphine in revision total hip arthroplasty.

This retrospective observational study was conducted between April 2023 and April 2024 at the Nuffield Orthopaedic Centre, Oxford, UK, a high‐volume revision arthroplasty centre. All adults undergoing revision total hip arthroplasty were included. Patients receiving continuous sedative or epidural opioid infusions postoperatively were not studied. Ethical approval was not required but Caldicott Guardian approval was obtained. The mode of anaesthesia was recorded, including intrathecal or regional anaesthesia. Doses of intrathecal morphine were then stratified into four dose groups (100 μg, 150 μg, 200 μg and 250 μg). Primary outcomes were total opioid consumption in the first 24 h (oral morphine equivalents) and highest pain scores at rest and movement during the first four 12‐h postoperative periods. Pain scores were recorded using routinely collected verbal descriptors on a 4‐point scale (0 = none; 1 = mild; 2 = moderate; 3 = severe), which is standard for our institution. Secondary outcomes were anti‐emetic, chlorphenamine and naloxone use in the first 24 h. Between‐group comparisons were performed with Mann–Whitney U or χ^2^ tests; multiple groups were compared using the Kruskal–Wallis test with post‐hoc Dunn's testing.

Of 201 patients undergoing revision total hip arthroplasty, 16 (79.6%) were not included due to postoperative epidural or intravenous opioid infusions, leaving 185 for analysis (Table 1). In total, 174 (94.1%) patients received concurrent general anaesthesia. Among the 51 (27.6%) patients who did not receive a spinal, 32 (63%) received a peripheral nerve block and 13 (10%) received both spinal anaesthesia and a block. The most common blocks were the fascia iliaca (n = 24) and femoral nerve (n = 13) blocks.

**Table 1:** Patient characteristics and treatments received. Values are mean (SD), number or median (IQR [range]).

	No spinal	Spinal, no intrathecal morphine	Intrathecal morphine 100 μg	Intrathecal morphine 150 μg	Intrathecal morphine 200 μg	Intrathecal morphine 250 μg
	n = 51	n = 27	n = 25	n = 11	n = 32	n = 37
Age; y	72 (13.3)	75 (9.8)	72 (14.3)	74 (17.2)	71 (12.8)	66 (14.2)
Sex; female	27	9	13	3	19	15
ASA physical status						
1	2	0	1	0	1	2
2	14	11	7	5	16	24
3	31	12	16	5	13	11
4	4	4	1	1	2	0
BMI; kg.m^‐2^	28.8 (5.4)	26.8 (4.3)	27.1 (7.7)	26.5 (6.5)	27.0 (5.0)	29.0 (5.7)
Pre‐operative opioids	19	7	6	3	5	11
Chlorphenamine	4	2	0	1	1	3
Anti‐emetic	11	4	6	1	12	12
Naloxone	0	0	0	0	1	1
Median worst pain score on movement (0–3)						
0–12 h	2 (1–3 [0–3])	2 (0–3 [0–3])	0 (0–1 [0–3])	0 (0–1 [0–2])	0 (0–1 [0–3])	0 (0–1 [0–3])
12–24 h	2 (1–2 [0–3])	1 (1–2 [0–3])	1 (0–1 [0–2])	0 (0–2 [0–3])	1 (0–2 [0–3])	1 (0–1 [0–3])
24‐h oral morphine equivalents; mg	63 (23–113 [0–1102])	53 (17–106 [0–880])	5 (0–20 [0–438])	10 (0–26 [0–40])	13 (0–40 [0–120])	10 (0–20 [0–990])

Intrathecal anaesthesia was administered to 134 patients (72.4%), of whom 107 (79.9%) received intrathecal morphine at a median (IQR [range]) dose of 200 (138–250 [75–250]) μg. The intrathecal morphine dose chosen at the discretion of the responsible anaesthetist was not associated with age, BMI, ASA physical status, or pre‐operative opioid use. Two patients received atypical doses (75 μg and 125 μg) and were not included in dose response analyses.

There was no significant difference in opioid consumption or pain scores between the 51 patients who did not have spinal anaesthesia and the 27 patients having spinal anaesthesia with no intrathecal opioid. Adding intrathecal morphine significantly reduced median (IQR [range]) 24‐h oral morphine equivalents compared with spinal anaesthesia without morphine (10 (0–21 [0–990]) mg vs. 53 (17–106 [0–880]) mg, p < 0.001). The intrathecal morphine dose was not associated with statistically significant differences in opioid use or pain scores (Table 1). Two patients who received intrathecal morphine, both ASA physical status grade 3 and given doses of 200 μg and 250 μg, required naloxone for bradypnoea.

Our experience in a high‐volume centre for revision total hip arthroplasty confirms that spinal anaesthesia with intrathecal morphine was associated with reduced postoperative 24‐h opioid consumption and lower pain scores compared with spinal anaesthesia without intrathecal morphine (during the first 24 h) and with no spinal anaesthesia (in the first 36 h). This mirrors evidence from primary total hip arthroplasty [4]. However, doses > 100 μg provided no additional analgesic benefit. Two cases of opioid‐induced ventilatory impairment requiring naloxone occurred with doses > 100 μg, suggesting a potential safety concern. Evidence from primary total hip arthroplasty similarly supports 100 μg as an effective dose with fewer adverse effects [5].

Opioid‐induced ventilatory impairment remains the most serious complication of intrathecal morphine and may be more likely in patients who are older and comorbid undergoing revision arthroplasty surgery. As revision procedures become more common, it is essential to standardise safe and effective analgesic strategies [4].

This study has several strengths. Despite revision surgery being uncommon, our large inclusive dataset reflects real‐world practice across a broad, unselected population, enhancing external validity. Limitations include heterogeneity in indications and techniques; assessment of short‐term endpoints; and pain scoring using a 0–3 scale which, whilst standard for our institution, is less common in the literature.
